# A scoping review of the views and experiences of health professionals in the Middle East and North Africa regions regarding social media use

**DOI:** 10.1080/20523211.2026.2647042

**Published:** 2026-06-19

**Authors:** Maan Ababneh, Derek Stewart, Scott Cunningham, Sarah Pedersen, Zachariah Nazar

**Affiliations:** a School of Pharmacy, Applied Sciences and Public Health, Robert Gordon University, Aberdeen, UK; b College of Pharmacy, QU Health, Qatar University, Doha, Qatar; c Graduate School, Robert Gordon University, Aberdeen, UK

## Abstract

**Background:**

Integrating social media into healthcare practice presents opportunities and poses key challenges for health professionals. While multiple reviews have been published, Western contexts receive the most attention, despite the distinct cultural and regulatory contexts that may shape social media use in other regions. The aim of this review was to scope the views and experiences of health professionals in the Middle East and North Africa region regarding social media use.

**Methods:**

Six databases were systematically searched from inception to November 2022 (updated search in March 2025). Studies published in English reporting the views and experiences of health professionals regarding social media use in 24 MENA countries were included. Two reviewers independently screened articles for selection, followed by data extraction and synthesis.

**Results:**

Thirty-eight studies were included; the majority adopted cross-sectional methodology (*n* = 36), with physicians the most studied group (*n* = 18) and mostly from Saudi Arabia (*n* = 22). Only two studies included theory in the development of data collection tools. Positive and negative views and experiences were reported along with the need for education and policies and guidance. Positive themes related to aspects of knowledge and skills, networking, patient communication, marketing, and perceptions of improved practice and enhanced patient care. Negative themes were less well reported and focused on data protection, social media being a distraction, and the perceived impact on well-being.

**Conclusions:**

Despite the substantial volume of existing studies, the evidence remains largely descriptive and lacking in theoretical grounding. More rigorous, in-depth research encompassing all health professional groups is needed to clarify the mechanisms through which social media influences practice. Such work is essential for informing robust policies, practical guidelines, and targeted educational strategies that can mitigate risks while maximising the professional and clinical benefits of social media use.

## Background

The rapid expansion of social media has transformed professional engagement across sectors, including healthcare, where it now serves as an important tool for clinical communication, education, and public health outreach (Al-Khalifa et al., 2021; El Daouk et al., 2020; Majid et al., 2020). Its capacity to disseminate health information, connect clinicians with patients, and facilitate telemedicine has been widely acknowledged. As a result, social media now supports multiple dimensions of healthcare delivery, from professional development and knowledge exchange to patient counselling and health promotion.

Although several reviews have examined health professionals’ use of social media, the majority originate from Western contexts. This has left gaps in understanding how social, cultural, and regulatory environments shape social media behaviour in other regions, particularly the Middle East and North Africa (MENA), where digital engagement patterns differ markedly from Western norms.

### Health professionals’ use of social media, opportunities and challenges

A broad body of research identifies multiple professional applications of social media, including health promotion, career advancement, peer networking, and research dissemination (Bahabri & Zaidan, 2021; Davoodi & Abed, 2003; Elkhayat et al., 2018). These platforms offer significant benefits for health professionals, healthcare institutions, patients, and the wider public. Evidence demonstrates that social media enhances continuing professional development, facilitates collaboration with colleagues and experts, and supports more responsive patient engagement (Bahabri & Zaidan, 2021; Davoodi & Abed, 2003; Elkhayat et al., 2018).

At the institutional level, social media has been leveraged for health surveillance, public health messaging, and policy influence (Alhayaza et al., 2021; Go-Globe, 2024). Several studies highlight its potential to strengthen health systems by enabling rapid information exchange, promoting evidence-based practice, and fostering community-level health awareness. Recent conceptual models have also mapped the individual, organisational, and technological factors influencing social media engagement, offering guidance for developing more effective health-focused digital content. For example, how clinicians’ digital literacy, institutional policies, and platform features shape the ways health professionals communicate with patients and disseminate health information online (Alhayaza et al., 2021).

However, this increased use of social media also introduces notable challenges. Concerns regarding e-professionalism, including reduced accountability, breaches of confidentiality, blurred professional – patient boundaries, and legal implications, are frequently reported (Ross & Myers, 2017). The proliferation of misinformation, particularly during the COVID-19 pandemic, has further highlighted vulnerabilities within digital environments (Glasdam et al., 2022; Peters et al., 2015; World Bank, 2022). These issues have prompted the development of national and institutional guidelines to support safe and ethical social media use among health professionals.

### Middle East and North Africa (MENA)

The MENA region, home to approximately 493 million people, is culturally, linguistically, and politically diverse, spanning from Morocco to Iran and including the six Gulf Cooperation Council (GCC) countries (World Bank, 2022). Social media plays a prominent role in public life within the region, with several MENA countries ranking among the highest globally in platform penetration and daily use (Go-Globe, 2024). Arabic has become the fourth most-used language on the internet, highlighting the region’s high levels of digital engagement (Alhayaza et al., 2021; Go-Globe, 2024).

While social media’s societal influence in MENA is well-recognised, including its role in communication, civic participation, and public discourse, far less is known about its implications for healthcare practice. Specifically, limited research has explored how health professionals in MENA use social media, perceive its impact, or navigate associated cultural and regulatory considerations.

### Objectives and definitions

The aim of this scoping review was to explore the views and experiences of health professionals in the Middle East and North Africa (MENA) region regarding their use of social media.

For the purposes of this review, social media refers to professional and public platforms (e.g. X/Twitter, LinkedIn, Facebook, Instagram, YouTube) used by health professionals for activities such as patient education, peer networking, dissemination of clinical information, and healthcare promotion.

### What this review adds to existing knowledge

Despite a growing international evidence base describing how health professionals use social media, existing reviews have largely addressed the topic at a global level or through an e-professionalism/medical professionalism lens, with syntheses frequently dominated by evidence from North America, Europe, and other high-income Western contexts (Farsi, 2021; Gholami-Kordkheili et al., 2013; Vukušić Rukavina et al., 2014). Consequently, the extent to which prevailing conclusions translate to the MENA region, where digital adoption, professional norms, and governance arrangements often differ, remains insufficiently characterised. This scoping review contributes new knowledge by providing the first region-wide evidence map of health professionals’ views and experiences of social media use across 24 MENA countries, synthesising the balance of reported benefits and harms, and explicitly identifying methodological and conceptual gaps that should guide future research and inform the development of locally responsive education, policy, and guidance.

## Methods

The scoping review followed Arksey and O'Malley's framework (Arksey & O’Malley, 2005) and Joanna Brigg's Institute standards for conducting scoping reviews (Peters et al., 2015). The review is reported according to the Preferred Reporting Items for Systematic Reviews and Meta-analyses extension for scoping review (Zricco et al., 2018).

### Inclusion and exclusion criteria

The inclusion criteria were described in terms of the *population, concept*, and *context* as follows: *population*: all health professionals; *concept*: views and experiences of health professionals regarding social media use; *context*: all 24 MENA countries, as defined by Davoodi and Abed.^20^ Studies conducted with students (undergraduate or postgraduate) were excluded, as were studies that did not report MENA data separately from other countries. Studies that did not focus on health professionals’ perspectives or lacked rigorous data collection methodologies were excluded. Letters, commentaries, perspectives, calls for change, and editorials were excluded. Studies published in English from database inception until the end of November 2022 were included. The search was re-run in March 2025 to retrieve more recent publications.

### Search strategy

The search was conducted in the American Psychological Association Database (APA), Cumulative Index to Nursing and Allied Health Literature (CINAHL), Embase, International Pharmaceutical Abstracts (IPA) and Medical Literature Analysis and Retrieval System (Medline/ PubMed). Search strings were developed for the key concepts of social media, health professionals and MENA, combined using Boolean operators (e.g. OR, AND) and truncation (Supplemental Material 1 provides full details of the search strategy). The reference lists of included papers were reviewed for further articles.

### Study selection

All citations were uploaded into RefWorks, and duplicates were removed. Titles and abstracts were independently screened by two reviewers. Relevant sources were retrieved in full, and their citation details were imported into Rayyan QCRI® (Ouzzani et al., 2016). The full text of each citation was independently assessed by two reviewers, with reasons for exclusion recorded. Discrepancies identified at any stage of the review were resolved through consensus and, where required, by adjudication from a third reviewer.

### Data extraction and synthesis

A data extraction tool was developed and piloted in Microsoft Excel to extract: authors, year, title, journal, country, aim, design, participants, number (response rate), setting, method, the theory used, data collection tool, key findings, the author stated strengths and weaknesses, and conclusion. Data extraction was undertaken by two reviewers independently; discrepancies identified were resolved through consensus and, where required, by adjudication from a third reviewer. An iterative thematic mapping approach was used to synthesise findings from the included studies following Arksey and O'Malley's framework (2005).^17^ Reviewers first familiarised themselves with the extracted data and generated initial codes, which were subsequently grouped into higher-order categories and refined through iterative team discussion. The high-level categories (i.e. *positive views and experiences, negative views and experiences*, *need for education*, and *need for policy and guidance*) were used as organising structures aligned with the review objectives, while the specific codes and themes within these categories were derived inductively from the data. To ensure consistency and analytic rigour, coding was calibrated on a subset of studies, a shared codebook was developed and refined, and discrepancies were resolved through consensus and, when necessary, consultation with a third reviewer.

## Results

### Data extraction

**Figure 1.**
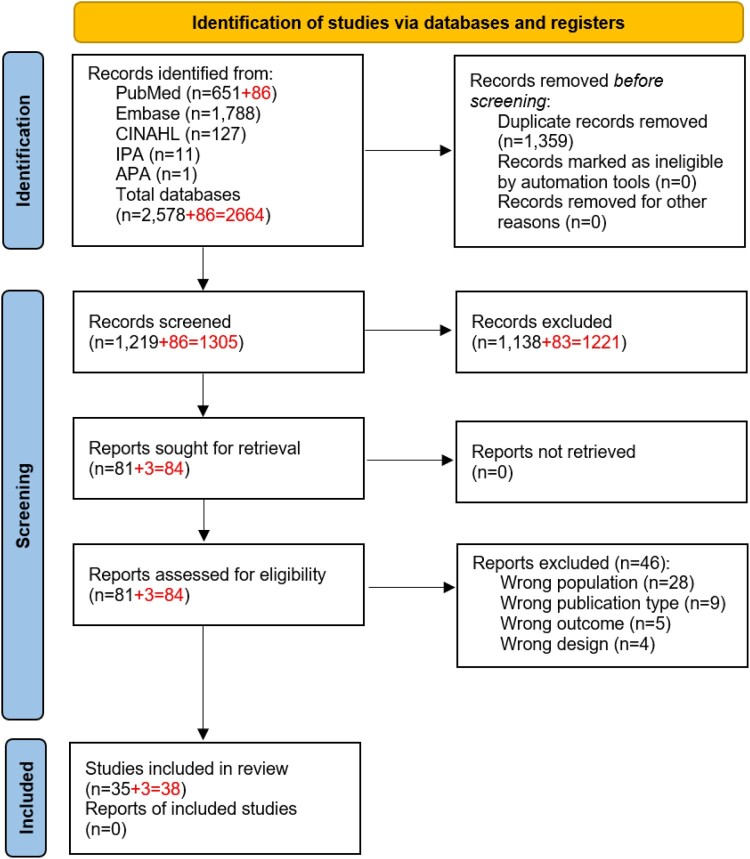
Scoping review PRISMA flow diagram.

This review included 38 articles (Figure 1). Over half of the studies were conducted in Saudi Arabia (*n* = 22), followed by Pakistan (*n* = 7), Lebanon, Egypt, United Arab Emirates (*n* = 2 each), and Iran, Turkey and Israel (*n* = 1 each) (Table 1). One study was conducted in the Arab world but did not specify the countries, and one reported data from 19 countries, including the Middle East. Most (*n* = 36) employed a cross-sectional methodology; two were qualitative, and three were mixed methods. In 18 studies, data were reported from single professional groupings of medical practitioners, 10 from dentists, 3 from nurses and 2 from pharmacists. A total of 9,257 participants were included, with the largest including 850 participants (cross-sectional survey of physicians, response rate of 6.8%) (El Daouk et al., 2020), and the smallest reporting qualitative data from 8 participants (Khan et al., 2019). In 20 studies, the setting was not specifically reported; in 18 studies specifying a setting, a wide range of governmental and private institutions were reported. Only two studies included theory in the development of data collection tools. Dailah and Naeem (2021) and Hazzam and Lahrech (2018) used the *Ability, Motivation, and Opportunity theory* and the *Technology Acceptance Model,* respectively. Most studies provided little detail on the stages of data collection tool development.

**Table 1.** Characteristics of included studies.

Authors, year, Country	Stated aim	Design, Participants, n, (response rate)	Setting
Abbasi et al., 2022. Pakistan	Investigate whether social media has affected the perceptions and demand for aesthetic dentistry.	Cross-sectional survey of 461 general dental practitioners	Sent via social media
Abdullah et al., 2022. Arab world	Explore how pharmacists in the Arab world are using Telegram for their professional purposes.	Cross-sectional survey of 267 (64%) pharmacists	Private or public pharmacies
Aboalshamat et al., 2019. Saudi Arabia	Investigate the usage, attitudes, and professionalism on social media among dental students and dentists in Saudi Arabia.	Cross-sectional survey of 438 dental students and 341 dentists/interns	Dental colleges
Afridi et al., 2020. Pakistan	Investigate the proportion of HCPs utilising social media for academic and professional purposes.	Cross-sectional survey of 512 (68.3%) doctors/ medical students	Hospital
Al Abbasi et al., 2021. Saudi Arabia	Evaluate the use of and beliefs about social media among Saudi ophthalmologists.	Cross-sectional survey of 293 (74.7%) ophthalmologists	Hospital
Al-Amad & Hussein 2021. 19 countries (incl. MENA)	Assessing the impact social media has on anxiety levels of dental healthcare workers whilst living through the COVID-19 pandemic.	Cross-sectional survey of 403 dental workers (91.8%) from MENA	Sent via social media
Alanzi & Al-Yami 2019. Saudi Arabia	Investigate physicians’ attitudes towards the use of social media for professional purposes in Saudi Arabia.	Cross-sectional survey of 235 (78.3%) physicians	Sent via social media
Alanzi et al., 2020. Saudi Arabia	Investigate HCPs’ perceptions of using LinkedIn for professional development in the Eastern Province of Saudi Arabia.	Cross-sectional survey of 181 HCPs	Sent via social media
Alanzi et al., 2021. Saudi Arabia	Investigate the perceptions of HCPs about the use of social media for healthy diet management in Saudi Arabia.	Cross-sectional survey of 156 (50.6%) HCPs	Sent via social media
Alasmary, 2022. Saudi Arabia	Explore the possibility of adopting Twitter as a means to improve health literacy among Saudi doctors.	Cross-sectional survey of 128 medical consultants	Not stated (active Twitter accounts)
Al-Dossary et al., 2022. Saudi Arabia	Investigate the use of social media in nursing education in Saudi Arabia.	Cross-sectional survey of 104 (34.7%) nurses	Ministry of Health and private hospitals
Alfozan et al., 2024. Saudi Arabia	Assess the role of social media in urology practice.	Cross-sectional survey of 170 (75.5%) urologists	Sent via social media
Alhayaza et al., 2021. Saudi Arabia	Investigate the percentage of the population that follows dermatologists on social media, and the reasons. Shed light on the activity of dermatologists on social media in Saudi Arabia and its effects on their practice.	Cross-sectional survey of 58 dermatologists	Not stated
Al-Khalifa et al., 2021. Saudi Arabia	Investigate dentists’ opinions towards social media use in daily practice and the expected limitations of its use in Saudi Arabia.	Cross-sectional survey of 392 (39.2%) dentists	Not stated
Alnufaiy et al., 2023. Saudi Arabia	Investigate perception and attitude toward social media use for professionalism and dental practice promotion among periodontists.	Cross-sectional survey of 300 (40.3%) periodontists	Sent via social media
Alqarni et al., 2021. Saudi Arabia	Assess the readiness of Saudi family medicine physicians to use social media in health promotion and explore their attitudes toward its use professionally.	Mixed methods. 136 (86.3%) family physicians, 11 interviews	Hospital-based primary care centres
Alshakhs & Alanzi, 2018. Saudi Arabia	Evaluate the perception of HCPs in Saudi Arabia towards the use of social media in health-care delivery.	Cross-sectional survey of 120 (83%) HCPs	All settings
Alsobayel, 2016. Saudi Arabia	Explore the use of social media networks for professional development among HCPs in Saudi Arabia.	Cross-sectional survey of 213 (9.2%) HCPs	Sent via social media
Alanzi et al., 2021. Saudi Arabia	Assess the role of social media in the training and continuing education of HCPs in Eastern Saudi Arabia.	Cross-sectional survey of 346 (72%) HCPs	Sent via social media
Azhar et al., 2022. Saudi Arabia	Explore how young urologists in Saudi Arabia are adopting social media as a learning tool.	Cross-sectional survey of 104 urologists/residents	Not stated
Bahabri & Zaidan, 2021. Saudi Arabia	Assess the perception of social media use for dental practice promotion, and associated professionalism amongst dental practitioners working in KSA.	Cross-sectional survey of 238 (64%) dental practitioners	Not stated
Barayev et al., 2021. Israel	Analyse the role of WhatsApp on the need for referrals to medical specialists and to compare the views of physicians regarding WhatsApp consultations.	Cross-sectional survey of 201 (59%) physicians	Defense Forces (Medical Corps)
Dailah & Naeem, 2021. Saudi Arabia	Provide a theoretical framework to explain how the use of SM can enhance the skills of HCPs and levels of organisational productivity.	Qualitative interviews with 30 nurses, 20 emergency doctors, surgeons	Government hospitals
Daniel et al., 2018. Lebanon	Describe how social media are regarded and utilised by practising and training physicians in Lebanon.	Cross-sectional survey of 238 (28.5%) physicians	Academic medical centre
Değirmencioğlu & Kılıçoğlu, 2024. Turkey	Evaluate orthodontists’ perceptions on the integration of social media in healthcare service delivery and marketing.	Cross-sectional survey of 2031 (18.6%) orthodontists	Email to Orthodontic Society members
El Daouk et al., 2020. Lebanon	Assess the behaviour of physicians using social media, identifying their awareness and defining communication breaches.	Cross-sectional survey of 850 (6.8%) physicians	Not stated
Elkhayat et al., 2018. Egypt	Define how cardiothoracic surgeons use social media and the effect of its use on surgical practice and patients’ management progress.	Cross-sectional survey of 83 (8.3%) orthopaedic surgeons	Not stated
Ghandhi et al., 2022. Pakistan	Evaluate the use of social media by dentists for professional purposes.	Cross-sectional survey of 404 (89.7%) dentists	Not stated
Hamasha et al., 2019. Saudi Arabia	Assess the dental practitioners’ use of social media, concerning demographic and social variables and the impact of social media use on dental practice.	Cross-sectional survey of 338 (77.2%) dental practitioners	Hospitals, centres, and clinics
Hazzam & Lahrech, 2018. United Arab Emirates	Explore the online behaviours of HCPs.	Cross-sectional survey of 203 (20.2%) physicians, pharmacists, & allied HCPs	Sent via social media
Ibrahim et al., 2020. United Arab Emirates	Assess the perception of patients and pharmacists in the United Arab Emirates about social media use in healthcare delivery.	Cross-sectional survey of 117 pharmacists	Not stated
Irfan et al., 2018. Saudi Arabia	To evaluate the utility of social media among family medicine residents and consultants.	Cross-sectional survey of 92 residents and 40 physicians (78%)	University participants
Justinia et al., 2019. Saudi Arabia	Assess the perceptions and usage of social media by orthopaedic surgeons in Jeddah, Saudi Arabia.	Mixed methods. 165 (51%) orthopaedic surgeons,	Medical centres, private or public
Kazemi et al., 2022. Iran	Explore nurses’ experiences of receiving social media and in-person education to integrate the findings into practice.	Qualitative interviews with 15 nurses	Hospitals
Khan et al., 2020. Pakistan	Assess if WhatsApp communication improves clinical knowledge, and explore the perception of its use among medical officers.	Mixed methods. 10 medical officers (survey), 8 (focus group)	Hospital
Majid et al., 2020. Pakistan	Investigate the effect of social networking site addiction on task distraction among nurses.	Cross-sectional survey of 413 (43%) nurses	Hospital
Siddiqui et al., 2020. Pakistan	Investigate the impact of using WhatsApp among orthopaedic surgeons.	Cross-sectional survey of 113 doctors	Orthopaedic departments
Younis et al., 2017. Egypt	Assess the use of social media by Egyptian female dermatologists. residents and their attitudes toward communicating with patients online.	Cross-sectional survey of 116 (92%) female dermatology residents	Conference attendees

### Data synthesis

Synthesis identified groupings of: positive views and experiences; negative views and experiences; need for education; and need for policies and guidance (Table 2).

**Table 2.** Synthesis of key findings of studies reporting the views and experiences of health professionals in MENA regarding social media.

Authors, year	Positive views and experiences						Negative views and experiences				Need for education	Need for policies and guidance
	Knowledge and skills	Networking	Patient communication	Marketing	Improved practice	Enhanced patient care	Data protection	Distracting	Wellbeing	Reduced patient care		
Abbasi et al., 2022			✓	✓								
Abdullah et al., 2022	✓	✓	✓		✓	✓						
Aboalshamat et al., 2019	✓	✓	✓				✓				✓	✓
Afridi et al., 2020					✓							
Al Abbasi et al., 2021			✓	✓	✓		✓	✓				✓
Al-Amad & Hussein, 2021	✓								✓			✓
Alanzi et al., 2020	✓	✓	✓	✓	✓	✓						
Alanzi et al., 2021	✓	✓										
Alanzi & Al-Yami, 2019	✓						✓					
Alasmary, 2022												✓
Al-Dossary et al., 2022	✓	✓									✓	
Alfozan et al., 2024	✓	✓	✓								✓	✓
Alhayaza et al., 2021					✓							
Al-Khalifa et al., 2021				✓							✓	
Alnufaiy et al., 2023.	✓		✓				✓				✓	✓
Alqarni et al., 2021												✓
Alshakhs & Alanzi, 2018	✓		✓	✓			✓			✓		
Alsobayel, 2016	✓				✓							
Alzain et al., 2021	✓					✓					✓	
Azhar et al., 2022	✓											
Bahabri & Zaidan, 2021				✓	✓		✓					✓
Barayev et al., 2021			✓				✓	✓				
Dailah & Naeem, 2021	✓	✓	✓									
Daniel et al., 2018							✓					✓
El Daouk et al., 2020		✓					✓			✓		✓
Değirmencioğlu & Kılıçoğlu, 2024			✓				✓					✓
Elkhayat et al., 2018			✓	✓								
Ghandhi et al., 2022	✓		✓				✓	✓		✓		
Hamasha et al., 2019	✓			✓	✓							
Hazzam & Lahrech, 2018		✓										
Ibrahim et al., 2020			✓			✓					✓	
Irfan et al., 2018					✓	✓						
Justinia et al., 2019			✓				✓					✓
Kazemi et al., 2022					✓			✓				
Khan et al., 2020	✓	✓	✓			✓						
Majid et al., 2020								✓	✓			
Siddiqui et al., 2020	✓				✓	✓						
Younis et al., 2017							✓			✓		

#### Positive views and experiences

##### Knowledge and skills

Sixteen studies described perceived benefits in terms of enhanced knowledge or skills. For example, several authors noted that social media provided accessible educational information and updates. In three studies, participants specifically linked social media engagement to perceived improvements in clinical or communication skills (Al-Amad & Hussein, 2021; Moorhead et al., 2013; Zricco et al., 2018).

##### Networking

Nine studies reported benefits related to networking, peer communication, or professional socialisation. In one mixed-methods study, authors used the term ‘professional socialization’ to describe these networking benefits (O’Connor et al., 2021).

##### Patient communication

Thirteen studies described perceived positive effects of social media on patient – provider communication. For instance, in some settings, health professionals viewed social media as a useful channel for patient education and outreach.

##### Marketing

Eight studies documented that participants considered social media an effective tool for marketing their professional services directly to patients. One survey in Pakistan reported that dentists believed social media advertising had increased demand for aesthetic dental procedures (Ghandhi et al., 2022).

##### Improved practice

In 13 studies, respondents believed that social media positively influenced their professional practice. For example, among dermatologists in Saudi Arabia (Khan et al., 2019), participants felt that use of social media had changed their clinical practice, although few concrete examples or objective outcome measures were provided.

##### Enhanced patient care

Seven studies described participants’ beliefs that social media improved aspects of patient care. However, none reported objective data on clinical outcomes; even in mixed-methods research, qualitative follow-up did not further investigate these perceptions (Rolls et al., 2016).

#### Negative views and experiences

##### Data protection

Participants in 11 studies expressed concern over data protection issues, including confidentiality and lack of privacy. Aboalshamat et al. reported that more than one-quarter of dentists surveyed in Saudi Arabia had considered posting patients’ information or photographs without seeking permission (Aboalshamat et al., 2019). Another study from Saudi Arabia reported participants’ concerns that inadvertent breaches of patient confidentiality could lead to ethical or legal challenges (Ibrahim et al., 2020). The authors concluded that these concerns would be heightened within the culture and customs in Saudi Arabia, but provided no further explanation. Younis et al. reported that these factors were associated with hesitation in fully embracing social media in Egypt (Younis et al., 2017).

##### Distraction

In 6 studies, social media use was considered a distraction. In one study in Pakistan, participants highlighted that social networking site addiction resulted in task distraction, with selected reports of professional envy, social anxiety, and rumination (Alshakhs & Alanzi, 2018). While participating in social media was considered valuable in supporting professional development in Iran, it was acknowledged that this consumed scarce human resources (Siddiqui et al., 2020).

##### Wellbeing

Moderate to severe anxiety was significantly associated with the frequency of social media use in 2 studies across a range of professional (Alshakhs & Alanzi, 2018; Greer et al., 2019).

##### Reduced patient care

Four studies reported the potential negative impact of social media use on reduced patient care due to patients opting out of traditional care, thus bypassing safety measures. There were also concerns over the dissemination of potentially inaccurate and harmful information (Dailah & Naeem, 2021; Grindrod et al., 2014; Hennessy et al., 2019; Ibrahim et al., 2020).

#### Need for education

In 6 studies, the need for health professionals’ education in the use of social media was highlighted. Education was largely referred to in general terms rather than specific areas of education. Authors alluded to actions such as focused campaigns and utilisation of professional development tools for social networking to maximise the effective use of social media platforms for both professional and personal (Aboalshamat et al., 2019; Alzain et al., 2021; Dailah & Naeem, 2021; Farsi, 2021; Ibrahim et al., 2020; Moorhead et al., 2013).

#### Need for policies and guidelines

Similarly, in 9 studies, the authors discussed the need for the development and implementation of policies and guidelines for health professionals on the use of social media; however, only four reported specific data. For example, one study noted that almost half of the participants in Saudi Arabia reported the absence of policies or guidelines, highlighting the need to regulate health information sharing and address professionalism, confidentiality and data protection concerns (Aboalshamat et al., 2019).

**Figure 2.**
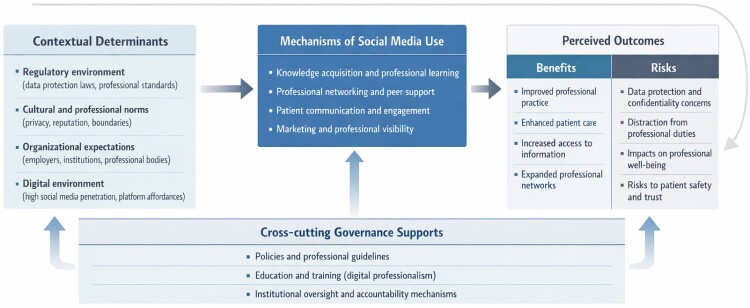
Conceptual model of health professionals’ social media use in the MENA region: relationships among context, mechanisms, outcomes, and governance supports.

To strengthen synthesis of the thematic findings, a conceptual model was developed to illustrate relationships among key themes identified in this review (Figure 2). The model integrates four components: (1) contextual determinants (regulatory environment, cultural and professional norms, and organisational expectations); (2) mechanisms of social media use (knowledge acquisition, professional networking, patient communication, and marketing); (3) perceived outcomes, including both benefits (improved professional practice and patient care) and risks (data protection concerns, distraction, and impacts on well-being); and (4) cross-cutting governance supports (policies, professional guidelines, and education and training) that mediate the balance between opportunities and risks. This visual synthesis provides an integrative framework for understanding how context and governance shape professional social media use in the MENA region.

## Discussion

### Key findings

This review synthesised findings from 38 studies examining health professionals’ use of social media across the MENA region. In contrast to prior global reviews dominated by evidence from North America and Europe (Farsi, 2021; Vukušić Rukavina et al., 2014), this scoping review provides a region-specific synthesis of views and experiences within MENA, where regulatory environments, professional norms, and patterns of digital adoption may shape both perceived benefits and risks (Gholami-Kordkheili et al., 2013; Labaki et al., 2021). By situating findings in this context, the review identifies evidence gaps and governance needs that are less visible in broader international syntheses.

The evidence base is dominated by research from Saudi Arabia and Pakistan, and by cross-sectional survey designs focused primarily on medical practitioners. Across studies, both positive and negative perceptions of social media use were identified, alongside a consistent call for enhanced education and clearer policy guidance. Positive perspectives centred on knowledge acquisition, professional networking, patient communication, marketing, and perceived improvements in practice or patient care. In contrast, negative perspectives were less comprehensively reported, highlighting concerns regarding data protection, distraction, professional well-being, and risks to patient care. Collectively, the findings reveal substantial heterogeneity in experiences but also point to two significant gaps in the literature: a notable underrepresentation of qualitative research capable of generating deeper contextual understanding, and a scarcity of studies informed by behavioural or theoretical frameworks that could strengthen explanatory power and guide intervention development.

### Strengths and limitations

This scoping review was comprehensive in scope and conducted in accordance with established methodological guidance, ensuring a transparent and systematic approach to evidence identification and synthesis. A key strength is the intentional focus on the MENA region, which allowed for the consolidation of perspectives shaped by distinct cultural, social, and professional contexts that may differ from those reported in Western settings. By mapping the breadth of existing research across multiple professional groups and countries, the review contributes a nuanced understanding of social media use within healthcare systems that are often underrepresented in global reviews.

Several limitations should be acknowledged. First, the inclusion criteria were restricted to studies published in English, which may have excluded relevant evidence published in Arabic or other local languages, particularly given the regional focus. Future reviews incorporating Arabic-language databases could yield a more complete picture of local practices and perceptions. Second, the included studies were unevenly distributed across the MENA region, with a substantial proportion originating from Saudi Arabia and Pakistan, while several countries had limited or no representation. This geographic concentration may reflect differences in research capacity, publication practices, and access to academic publishing across countries. As a result, the findings may be more reflective of contexts where research activity on digital health and social media is more established, potentially limiting the generalizability of the results across the wider MENA region. Thirdly, it was evident that many included studies provided limited information regarding the development, validation, and piloting of their data collection tools. This raises concerns about measurement robustness and may affect the reliability and interpretability of findings. Finally, the predominance of cross-sectional self-report surveys, combined with a scarcity of qualitative and theoretically informed studies, restricts the depth of insight into underlying behaviours, contextual influences, and causal mechanisms.

### Interpretation

Across included MENA studies, health professionals reported a recurring tension between the perceived benefits of social media for professional learning, networking, and patient engagement, and concerns regarding confidentiality, professional boundaries, reputational risk, and misinformation. While prior international syntheses have documented similar benefits and risks, they have largely provided global overviews without explicitly examining how regional governance arrangements shape these experiences (Giuffrida et al., 2024; Hennessy et al., 2019). To frame the interpretation, our findings can be understood using the Walt and Gilson health policy triangle (content, context, actors, and processes) (O'Brien et al., 2020; Walt et al., 2008; Zahidie et al., 2023). Within this framework, calls for clearer guidance on confidentiality, professional boundaries, and conflicts of interest reflect the ‘content’ dimension; regulators, employers, professional bodies, educators, and clinicians constitute key ‘actors’; ‘processes’ concern how guidance is developed and implemented; and ‘context’ includes legal norms, organisational cultures, and platform-driven dynamics. This framing highlights that the balance between opportunities and risks is shaped not only by individual behaviour but also by governance structures and institutional environments.

Several features of the MENA context appear to distinguish these findings from those reported in Europe, North America, and Australia. High social media penetration and widespread internet access, particularly in Gulf Cooperation Council countries, may increase opportunities for professional and patient-facing engagement. At the same time, evolving regulatory environments, including strengthened personal data protection laws, heighten the salience of confidentiality, consent, and professional accountability. Cultural and professional norms related to privacy and reputation may further amplify sensitivity to online conduct. However, few included studies operationalised regulatory or cultural factors, highlighting the need for qualitative and theory-informed research to better explain how context shapes professional social media use in the region.

Consistent with international literature, social media was widely perceived as facilitating communication with patients and networking with peers and experts (Farsi, 2021; Katz & Nandi, 2021; Ouzzani et al., 2016). Some studies in the MENA region sought to conceptualise these networking benefits, including through the Social Media Organisational Productivity model, which proposed that peer interaction may enhance organisational communication and productivity within public hospitals (Dailah & Naeem, 2021). Professional networking was also encouraged within existing guidance from medical governing bodies (Al-Khalifa et al., 2021). Studies further reported perceived knowledge advancement through social media use; however, concerns raised in international reviews regarding the reliability and credibility of educational content were rarely addressed in the included literature.

In contrast to international reviews in which marketing by health professionals was infrequently reported (Katz & Nandi, 2021; Ouzzani et al., 2016), this review identified a substantial number of marketing-related studies, particularly among dental practitioners (Abbasi et al., 2022; Al-Amad & Hussein, 2021; Farsi, 2021; Mate et al., 2017). This pattern may reflect the growth of the private healthcare sector in several MENA countries and increasing competition among providers (Hamm et al., 2013), especially within Gulf Cooperation Council settings (Yazbeck et al., 2017).

Despite frequent reports of perceived improvements in patient care and professional practice, nearly all evidence was based on self-reported perceptions, with limited specification of which aspects of care or practice were enhanced. Only one study reported benefits beyond clinicians’ perceptions (Abbasi et al., 2022). Similar limitations have been noted in international reviews, where objective evidence linking social media use to patient outcomes remains scarce (Farsi, 2021). These constraints highlight the methodological challenges of evaluating the direct impact of social media on clinical practice.

Many studies emphasised the need for clearer social media policies, professional guidelines, and targeted education. Gaps were identified in areas such as data protection and management of personal – professional boundaries online, reflecting both regional regulatory developments and persistent uncertainty among practitioners. These findings align with international guidance stressing the importance of supporting professional behaviour while recognising online risks (Al-Khalifa et al., 2021; Mayer et al., 2012). The prominence of guidance and training needs can also be interpreted through the Multiple Streams Framework, whereby policy change becomes more likely when problem recognition, feasible solutions, and supportive institutional climates converge (Béland & Howlett, 2016; Hoefer, 2022). This perspective helps explain why social media policies and guidelines emerge unevenly across professions and settings.

Notably, negative views and experiences were reported less frequently than positive ones. Concerns regarding data protection, distraction, and professional well-being were identified but may be underrepresented due to the predominance of survey-based designs and the limited use of qualitative or theoretically informed approaches. Previous reviews have similarly found that qualitative studies are particularly important for uncovering negative experiences and contextual complexities (Vukušić Rukavina et al., 2014). Cultural influences were also rarely examined directly, reflecting limitations in survey instruments and the narrow conceptual scope of much of the existing literature.

### Further research

Taken together, these findings imply that strengthening ‘digital professionalism’ in MENA requires coordinated action across the policy triangle. At the content level, guidance should explicitly address confidentiality, boundary management, professional representation, and conflicts of interest, aligning expectations across clinical and academic contexts. At the actor level, regulators, professional associations, and employers should clarify roles and accountability for staff training and incident response. At the process level, implementation strategies (orientation, refresher training, auditing/feedback, and fair escalation pathways) are needed to translate written guidance into consistent practice. Finally, the context dimension highlights the need to tailor interventions to local regulatory realities and organisational cultures, while remaining responsive to evolving platform affordances and misinformation pressures.

Future research should move beyond descriptive surveys to incorporate qualitative, longitudinal, and theory-informed approaches that can better explain behavioural drivers, cultural influences, and organisational factors shaping social media use. Such methodological diversification is essential for generating evidence that can inform policy development, guide the safe integration of social media into professional practice, and evaluate its potential impact on healthcare delivery and patient outcomes.

## Conclusions

This scoping review identified a large number of studies reporting the positive and negative views and experiences of health professionals in the MENA region relating to social media use. Two key gaps were apparent: a scarcity of qualitative research that can illuminate contextual and cultural influences, and limited use of theoretical frameworks to explain behaviour or guide intervention development. Addressing these gaps is essential for strengthening the evidence base and supporting the development of context-appropriate policies, guidelines, and educational initiatives. As social media continues to shape healthcare communication and professional practice in the region, more rigorous and theoretically informed research will be critical to ensuring its safe, effective, and professionally responsible use.

## Supplementary Material

Supplmentary Material 1.docx
