# Supplementary material for: A scoping review of the views and experiences of health professionals in the Middle East and North Africa regions regarding social media use
Source: J Pharm Policy Pract. 2026 Jun 19;19(1):2647042. doi: 10.1080/20523211.2026.2647042 (PMC13288534; doi:10.1080/20523211.2026.2647042)
Supplement: Supplmentary Material 1.docx [file JPPP_A_2647042_SM4348.docx]

**Supplementary Material 1: Search strings applied to electronic databases**
